# The prevalence of sarcopenia and relationships between muscle and bone in ageing West‐African Gambian men and women

**DOI:** 10.1002/jcsm.12341

**Published:** 2018-09-16

**Authors:** Ayse Zengin, Landing M. Jarjou, Ann Prentice, Cyrus Cooper, Peter R. Ebeling, Kate A. Ward

**Affiliations:** ^1^ Department of Medicine, School of Clinical Sciences, Faculty of Medicine, Nursing and Health Sciences Monash University, Monash Medical Centre Clayton VIC Australia; ^2^ MRC Elsie Widdowson Laboratory Cambridge UK; ^3^ MRC Unit The Gambia Keneba The Gambia; ^4^ MRC Lifecourse Epidemiology Unit University of Southampton Southampton UK; ^5^ National Institute for Health Research (NIHR) Musculoskeletal Biomedical Research Unit University of Oxford Oxford UK

**Keywords:** Sarcopenia, Muscle, Bone, Ethnicity, Ageing, Jumping mechanography

## Abstract

**Background:**

The rapidly rising ageing population in low and middle‐income countries (LMIC) will lead to a concurrent increase in musculoskeletal diseases. Sarcopenia is a disease caused by progressive loss of skeletal muscle mass and strength, leading to adverse outcomes including frailty, falls, fractures, and premature mortality. We investigated the prevalence of sarcopenia, assessed the suitability of current diagnostic guidelines and explored muscle–bone relationships in ageing men and women from rural Gambia.

**Methods:**

A total of 249 women and 239 men aged 40–75+ years were recruited. Body composition was measured using dual energy X‐ray absorptiometry. Comparisons of the Foundations for the National Institutes of Health (FNIH) and European Working Group On Sarcopenia (EWGSOP) definitions of sarcopenia to define prevalence and to identify poor physical capability were determined. Functional ability was assessed by jumping mechanography to calculate lower limb muscle force and power; grip strength was assessed by a hand dynamometer. Peripheral quantitative computed tomography was used to assess muscle–bone relationships.

**Results:**

The prevalence of sarcopenia in Gambian men and women significantly varied depending on the definition used; in men 20% and 19% and in women 45% and 10% for FNIH and EWGSOP, respectively. The FNIH appendicular lean mass cut‐off had greatest sensitivity and specificity in identifying low functional ability in Gambian adults. Muscle force was positively associated with measures of tibial bone size, strength, and mineral content.

**Conclusions:**

The variation in the prevalence of sarcopenia depends on the definition used and highlights the importance of measuring functional capability across ethnic populations.

## Introduction

Musculoskeletal disease is a major contributor to the global non‐communicable disease (NCD) burden.^1^ Recently, the Global Burden of Disease study showed that the disability‐adjusted life years lost to musculoskeletal diseases was 119 million worldwide.^2^ To date, health priorities in low and middle‐income countries (LMICs) have mostly focused on infectious disease; as such, NCDs are underrepresented in national health agendas. With the current social, economic, and environmental transition in LMICs, diseases of older age, namely, sarcopenia and osteoporosis, are becoming increasingly prevalent. It is also important to note that cardiometabolic disease and HIV, both major burdens in LMICs, are co‐morbidities associated with musculoskeletal disease.

Sarcopenia is characterized by progressive loss of skeletal muscle mass and/or strength, thereby increasing the risk of adverse outcomes such as frailty, falls, fractures, and mortality.^3^, ^4^, ^5^, ^6^ Only recently, sarcopenia has been recognized as a disease entity (ICD‐10‐CM).^7^ Sarcopenia is associated with osteoporosis—both are chronic conditions reducing functional ability, balance, daily locomotion, and mobility. The most widely used definitions of sarcopenia from the Foundation for the National Institutes of Health (FNIH) and the European Working Group on Sarcopenia in Older People (EWGSOP) include cut‐points for appendicular lean mass and functional ability (grip strength and/or gait speed). However, neither are fully accepted, as the varying approaches affect the estimated prevalence, which ranges from 10% to 30%, and are predominantly based on Caucasians from high income countries (HIC). Little is known about whether these definitions are suitable to determine the prevalence of sarcopenia across populations, where known differences in proportions and amounts of fat and lean tissue exist, which are likely to change the appropriateness of selected cut‐offs.^8^, ^9^

Studies from HICs have shown that low muscle power, a measure of functional ability, is associated with increased fall risk in the ageing population,^10^, ^11^ where falls are the main cause of hip fracture.^12^ Muscle power is the product of force and velocity, facilitating muscles to rapidly generate force and thereby enabling mobility and balance.^13^, ^14^, ^15^ Muscle force and muscle power both decline with age, with power declining at a greater rate than force^16^ and more rapidly in men than in women.^17^ The variance in functional capability is explained more by muscle power than muscle force^18^; however, it is muscle force that gives a measure of load to the bone and is used to study muscle–bone relationships. Through remodelling, bone strength changes to alter the bone size and the distribution of bone mineral content (BMC) in response to strains generated through forces during a muscle contraction. These adaptations maintain bone strength, prevent damage, and ultimately fracture. Therefore, muscle power and force are both important in the maintenance of musculoskeletal health.

It is yet unknown whether the existing definitions of sarcopenia are able to robustly determine the prevalence of sarcopenia across populations and ethnic groups, particularly in LMICs. Secondly, whether or not the traditional measure of grip strength better identifies low functional ability than lower limb muscle power or force in Gambian men and women needs to be resolved. Therefore, this study aimed to determine the prevalence of sarcopenia as defined by FNIH and EWGSOP in ageing Gambian men and women and whether these discriminated poor functional capacity in this population. Secondly, to assess muscle–bone relationships using measures of muscle area, density, and bone by peripheral quantitative computed tomography and measures of muscle function using jumping mechanography.

## Materials and methods

### Participants and study design

The Gambian Bone and Muscle Ageing Study (GamBAS) is a longitudinal prospective observational study investigating musculoskeletal ageing in men and women from a rural region of The Gambia.^19^ Briefly, men and women aged 40–75+ years were recruited from the rural villages in the Kiang West region. Frail, pregnant, or lactating individuals were excluded. Stratified sampling was used to ensure recruitment of equal numbers of men and women in each of the eight, 5‐year age bands, namely, 40–44, 45–49, 50–54, 55–59, 60–64, 65–69, 70–74, and 75 years and over; in total, 249 women and 239 men were recruited. The current report is based on the baseline measurements from this study. The study received ethical approval by the joint Gambia Government/MRC The Gambia Ethics Committee. Written informed consent was obtained from all participants, and all procedures were carried out in accordance with the Declaration of Helsinki.^20^ All measures were assessed at a single research visit. Weight (kg) was measured using electronic scales, and standing height (cm) was measured using a stadiometer. A general health questionnaire collected data on activities of daily living (e.g. gardening, occupation, and farming).

### Dual‐energy X‐ray absorptiometry

Body composition was assessed by DXA^21^ (GE Lunar Prodigy, Waltham, MA, USA) software version 10. Body composition measures were whole body lean mass (kg) and whole body fat mass (kg). Appendicular lean mass (aLM) was calculated as the sum of the lean mass of the arms plus legs (kg); two scans were excluded: one due to a participant with leprosy and another due to movement artefact.

### Definition of sarcopenia

The proportion of sarcopenic men and women was calculated using the different classifications: the Foundation for the National Institutes of Health (FNIH criteria of appendicular lean mass of <19.75 kg in men and <15.02 kg in women were defined as sarcopenic.^22^ The European Working Group on Sarcopenia in Older People (EWGSOP) criteria of aLM adjusted for height squared combined with low grip strength (men: <7.25 kg/m^2^ and <30 kg; women: <5.45 kg/m^2^ and <20 kg).^4^

### Grip strength

Grip strength (kg) was measured using a hand dynamometer (Jamar Hand Dynamometer, Patterson Medical, Bolingbrook, IL, USA).^23^ The individual was seated in an upright position with the arm supported on the armrest of the chair with the wrist in a neutral position and the thumb facing upwards. Participants were instructed to exert maximal force. For each individual, the first of four measurements was regarded as a practice, and the maximum force (kg) in the following three measurements was recorded as the participant's grip strength.

### Jumping mechanography

To assess lower limb muscle force and power, a Leonardo Mechanography Ground Reaction Force Platform (Leonardo software version 4.2; Novotec Medical GmbH) was used.^24^ Participants were asked to perform three tests: a single two‐leg countermovement jump (s2LJ), a multiple one‐leg hopping test (m1LH), and a chair rise test (CRT). Participants performed each test three times. Briefly, for the s2LJ: individuals were asked to jump, bending their knees to jump as high as possible, and the jump with highest height was used for analysis. The Esslinger Fitness Index (EFI) compares the relative power of a participant to the average of an age and sex‐matched reference group.^25^ For the m1LH: individuals were instructed to hop on the dominant forefoot with a stiff knee and without the heel touching the ground, approximately 10 times. Those individuals who were too frail or did not feel comfortable to perform the s2LJ or m1LH were classified as ‘unable’ to complete the test. In total, there were 362 participants who performed the s2LJ/m1LH test. For the CRT: individuals were asked to stand from a chair, which was anchored to the Leonardo Mechanography Ground Reaction Force Platform (as above) without using their arms; those who were unable to do a single chair stand were classified as ‘unable’ to complete the test (*n* = 5). Individuals who were able to complete the single chair stand were asked to repeat the chair rise test five times as quickly as possible. The intra‐ and inter‐rater reliability of jumping mechanography has been reported^26^, ^27^ with a CV of 0.3–0.6% in 10 healthy adults.

### Peripheral quantitative computed tomography

Peripheral QCT (pQCT) measurements were made at the tibia using a Stratec XCT‐2000 scanner (Stratec, Pforzheim, Germany), software version 6.20c. All measurements were taken in the non‐dominant limb. Leg length was defined as the distance from the most proximal edge of the medial malleolus to the intercondylar eminence. Measurements were taken at the 38% and 66% of the limb length (measured with tape measure). Cross‐sectional area (CSA; mm^2^), cortical area (Ct.Area; mm^2^), cortical bone mineral content (Ct.BMC; mg/mm), stress strain index, which is a measure of the bones torsional strength (SSI; mm^3^), and cross‐sectional moment of inertia (CSMI; mm^4^) were measured at the 38% tibia. Cross‐sectional muscle area (CSMA; mm^2^) and muscle density (mg/cm^3^) were measured at the 66% tibia. All scans were analysed using separation mode 1; threshold = 710 mg/cm^3^ for the 38% site, threshold = 280 mg/cm^3^ for SSI, and threshold = 40 mg/cm^3^, filter F03F05 for CSMA. Muscle density was assessed using threshold = 100 mg/cm^3^, filter F03F05. For this analysis, we excluded scans that were affected by excessive motion that had distorted the image.^28^ The range of coefficient of variation (CV) of duplicate measurements of the tibia for precision of muscle and bone variables in 62 Gambian adults was 1–3%.

### Data analysis

All analyses were performed in Stata, Version 14.0 (StataCorp, College Station, TX, USA), and results were considered statistically significant at *P* < 0.05. Descriptive statistics were used to describe participant characteristics and are presented as mean ± standard deviation (SD) and categorical variables as frequency with per cent. Between‐group differences in the participant characteristics were tested with a one‐way ANOVA and with a χ^2^ test for categorical variables.

The agreement between FNIH and EWGSOP defined sarcopenia and functional measures was tested. Receiver operating characteristic (ROC) analyses were used to determine the area under the curve (AUC), the sensitivity, and the specificity for each method to discriminate between sarcopenic individuals who had low versus normal functional ability as defined by the following cut‐offs:

- FNIH definition of low grip strength: <26 kg in men and <16 kg in women;
- EWGSOP definition of low grip strength: <30 kg in men and <20 kg in women.

For the measures of muscle force and power, the sex‐specific 20th percentile was chosen as the cut‐off point for s2LJ power, force, and m1LH force.^4^, ^29^

- Low s2LJ muscle power: <0.97 kW in men and <0.66 kW in women;
- Low s2LJ muscle force: <1.17kN in men and <0.93kN in women;
- Low m1LH muscle force <1.14kN in men and <0.83kN in women.

Univariate odds ratios with 95% confidence intervals were then calculated to describe how well the sarcopenic definitions predicted poor physical capability in this population. We tested the differences between the AUC to see which sarcopenia definition better detected low muscle function and reported significant differences as *P* < 0.05. Participants who were considered “unable” to perform the s2LJ and the m1LH, and who didn't have aLM data were excluded from the ROC analyses (*n* = 126), with 362 participants included in the final analyses. We tested whether there were differences in body habitus, bone parameters and CRT parameters between those who were “able” and “unable” to perform the jumping tests. Pearson correlation was used to test whether there were associations between CRT relative power vs. s2LJ power in the ‘able’ group; we then tested associations between CRT relative power and grip strength in all participants.

We used linear regression to investigate the relationship between bone measures (dependent variable) and s2LJ/m1LH muscle force (independent variable), with adjustments for weight and height; a muscle force*sex interaction was tested using a Wald test. The muscle force*sex interaction term was removed when non‐significant and the *P*‐value for the main effect of muscle force was reported. Bone measures were log transformed to normalize distributions.^30^ We investigated the effect of age on the relationship between bone measures and muscle force with a muscle force*age interaction; these analyses were all non‐significant and were not included. Similarly, age as a main predictor was non‐significant and therefore was not included. Values are presented as beta coefficients expressed as a per cent change for every 1 kN change in muscle force with 95% confidence intervals. To facilitate the visualization of the results, bone parameters were transformed into z‐scores (per SD).

## Results

*Table* 1 shows the participant characteristics for men and women. The prevalence of sarcopenia was similar in men (~20%) irrespective of the classification that was used. However, there was a difference in the prevalence of sarcopenia in women depending on the definition (*Table* 1). According to the FNIH definition, 45% of women were sarcopenic of which 68% were aged over 60 years, in contrast to 10% with the EWGSOP definition of which 85% were over 60 years of age (*Table* 1).

**Table 1:** Participant characteristics

	Men	Women	*P*‐value
Body habitus	*n* = 239	*n* = 249	
Age (year)	60·8 ± 12.3	61.1 ± 12.5	0.798
Body weight (kg)	59.9 ± 10.3	54.7 ± 10.3	**<0.0001**
Height (cm)	169.2 ± 7.0	157.8 ± 6.0	**<0.0001**
BMI (kg/cm^2^)	20.9 ± 3.1	21.9 ± 3.7	**0.001**
	*n* = 235	*n* = 242	
Whole body fat mass (kg)	8.3 ± 6.0	17.1 ± 7.4	**<0.0001**
Whole body fat percent (%BW)	13.0 ± 7.3	30.0 ± 8.0	**<0.0001**
Whole body lean mass (kg)	49.0 ± 6.2	35.0 ± 4.3	**<0.0001**
	*n* = 238	*n* = 248	
Appendicular lean mass (kg)	22.9 ± 3.6	15.6 ± 2.4	**<0.0001**
Currently working in garden (%, freq)	81 (192)	87 (210)	0.097
Prevalence of sarcopenia	*n* = 238	*n* = 248	
FNIH aLM (*n*, %)	48 (20)	112 (45)	**<0.0001**
<60 years	9 (19)	36 (32)	**<0.0001**
>60 years	39 (81)	76 (68)	**<0.0001**
EWGSOP (*n*, %)	45 (19)	26 (10)	**0.009**
<60 years	8 (18)	4 (15)	**0.001**
>60 years	37 (82)	22 (85)	**<0.0001**
38% Tibia	*n* = 218	*n* = 228	
Ct. BMC (mg/mm)	364.5 ± 51.9	244.1 ± 52.5	**<0.0001**
Ct. Area (mm^2^)	301.1 ± 39.6	207.1 ± 37.4	**<0.0001**
CSA (mm^2^)	453.3 ± 56.9	359.5 ± 48.0	**<0.0001**
CSMI (mm^4^)	16063.8 ± 4509.8	9062.4 ± 2578.4	**<0.0001**
SSI (mm^3^)	1983.2 ± 332.2	1299.5 ± 262.0	**<0.0001**
66% Tibia	*n* = 214	*n* = 223	
CSMA (mm^2^)	5844 ± 1042	4541 ± 872	**<0.0001**
	*n* = 201	*n* = 227	
Muscle density (mg/cm^3^)	70.5 ± 2.5	69.2 ± 3.2	**<0.0001**
s2LJ	*n* = 208	*n* = 189	
Force (kN)	1.5 ± 0.4	1.2 ± 0.3	**<0.0001**
Relative force (N/kg)	24.4 ± 4.3	21.5 ± 3.0	**<0.0001**
Power (kW)	1.8 ± 0.9	1.0 ± 0.4	**<0.0001**
Relative power (W/kg)	28.5 ± 12.0	18.6 ± 6.3	**<0.0001**
Force efficiency (%)	63.7 ± 15.8	63.9 ± 14.9	0.884
Force efficiency z‐score (SD)	−3.6 ± 1.6	−3.6 ± 1.5	0.803
EFI z‐score (SD)	−2.1 ± 1.3	−2.2 ± 0.9	0.195
Maximum velocity (m/s)	1.6 ± 0.5	1.2 ± 0.3	**<0.0001**
m1LH	*n* = 185	*n* = 185	
Force (kN)	1.4 ± 0.4	1.0 ± 0.3	**<0.0001**
Relative force (N/kg)	2.4 ± 0.4	2.0 ± 0.3	**<0.0001**
Force z‐score (SD)	−3.0 ± 1.4	−4.4 ± 1.0	**<0.0001**
CRT	*n* = 237	*n* = 246	
Relative force (N/kg)	1.4 ± 0.3	1.3 ± 0.3	**0.021**
Relative power (W/kg)	8.8 ± 2.9	6.1 ± 1.8	**<0.0001**
Time per test (s)	5.1 ± 1.7	5.2 ± 1.8	0.632

All values are mean ± SD. Bold indicates *P* < 0.05. The proportion of participants classified as sarcopenic were separated into less than 60 years and greater than 60 years of age.

BMC, bone mineral content; BMI, body mass index; BW, body weight; CRT, chair rise test; CSA, cross‐sectional area; CSMA, cross‐sectional muscle area; CSMI, cross‐sectional moment of inertia; Ct, cortical; EFI, Esslinger Fitness Index; EWGSOP, European Working Group on Sarcopenia in Older People; m1LH, multiple one leg hop; SD, standard deviation; SSI, stress strain index; s2LJ, single two‐legged jump.

The agreement between the two sarcopenia cut‐points from lean mass measurements to predict low grip strength, muscle power, and force is presented in *Table* 2. The FNIH aLM classification had better sensitivity and specificity compared with the EWGSOP definition for all functional measures for both men and women. The FNIH classification had higher sensitivity in women than in men (range 62–76% and 37–50%, respectively), while specificity was slightly higher in men than in women (range 87–94% and 60–70%, respectively).

**Table 2:** Comparisons of sarcopenia cut‐points in identifying low grip strength, muscle power, and muscle force in men and women

		Men				Women			
		Sensitivity (%)	Specificity (%)	AUC	OR (95% CI)	Sensitivity (%)	Specificity (%)	AUC	OR (95% CI)
FNIH aLM	FNIH GS^a^	43.9	87.3	0.656	5.4 (2.7, 10.6)	70.5	60.3	0.654	3.6 (1.8, 7.3)
	EWGSOP GS^a^	36.5	92.5	0.645	7.1 (3.4, 15.0)	62.5	67.4	0.649	3.4 (2.0, 5.8)
	s2LJ power^b^	46.4	91.1	0.688	8.9 (3.6, 22.0)	70.0	67.3	0.687	4.8 (2.1, 11.1)
	s2LJ force^b^	50.0	92.3	0.711	11.9 (4.8, 29.8)	75.9	68.2	0.721	6.8 (2.8, 16.5)
	m1LH force^b^	48.6	93.9	0.713	14.6 (5.8, 36.7)	74.3	69.7	0.720	6.7 (2.9,5.1)
EWGSOP	FNIH GS^a^	45.6	89.5	0.676	7.2 (3.6, 14.4)	20.5	91.7	0.561	2.8 (1.2, 6.8)
	EWGSOP GS^a^	39.4	97.0	0.682	21.2 (7.5, 59.0)	21.2	97.2	0.592	9.4 (3.3, 26.9)
	s2LJ power^b^	39.3	91.1	0.652	6.6 (2.6, 16.6)	23.3	95.2	0.593	6.1 (2.0, 18.3)
	s2LJ force^b^	50.0	93.5	0.718	14.5 (5.6, 37.4)	27.6	95.9	0.618	9.0 (3.0, 27.5)
	m1LH force^b^	48.6	95.3	0.720	19.1 (7.2, 50.6)	20.0	95.1	0.575	4.8 (1.6, 14.3)

AUC, area under the curve; CI, confidence interval; EWGSOP, European Working Group on Sarcopenia in Older People; FNIH, Foundation for the National Institutes of Health; GS, grip strength; OR, odds ratio.

a indicates *n* = 238 for men and *n* = 248 for women.

b indicates *n* = 185 for men and *n* = 177 for women.

The AUCs from the receiver operating characteristic analyses showed that FNIH aLM had moderate‐good ability to discriminate those with low versus normal s2LJ force in men (AUC = 0.71) and women (AUC = 0.72), as well as low versus normal s2LJ power in men (AUC = 0.69) and women (AUC = 0.69). The AUCs for EWGSOP to discriminate between those with a low versus normal functional ability in men and women (across all five muscle function tests) reflect insufficient discriminative power (*Table* 2). In women, the FNIH compared with the EWGSOP definition significantly better detected low s2LJ force (*P* = 0.004), m1LH force (*P* = 0.002), FNIH defined low grip strength (*P* = 0.02), and displayed a trend in s2LJ power (*P* = 0.059), while there were no differences between the different functional tests in men. The participants that were ‘unable’ to perform the m1LH/s2LJ tests were older, weighed less, had lower aLM, and lower bone parameters at the 38% tibia compared with the participants who were ‘able’ to perform these tests (*Table* 3). CRT time was greater in the ‘unable’ group in line with lower relative CRT power and grip strength (*Table* 3). CRT relative power and s2LJ power were significantly correlated in the ‘able’ group (*R* = 0.69, *P* < 0.0001). Similarly, CRT relative power and grip strength were significantly correlated in all participants (*R* = 0.63, *P* < 0.0001).

**Table 3:** Sub‐analysis of participants who were ‘able’ and ‘unable’ to perform m1LH and s2LJ tests

	*n*	Able	*n*	Unable	*P*‐value
Body Habitus					
Sex (*n*, M/F)	362	185/177	126	54/72	0.111
Age (year)	362	57.1 ± 10.6	126	72.0 ± 10.5	**<0.0001**
Body weight (Kg)	362	58.4 ± 10.6	126	54.0 ± 10.0	**0.0001**
Height (cm)	362	164.3 ± 8.3	126	160.6 ± 9.1	**<0.0001**
BMI (kg/cm^2^)	362	21.6 ± 3.5	126	20.9 ± 3.1	0.052
Whole body fat mass (kg)	358	12.7 ± 8.2	119	12.8 ± 7.5	0.913
Whole body fat per cent (%BW)	358	22.3 ± 12.2	119	24.1 ± 11.7	0.148
Whole body lean mass (kg)	358	42.9 ± 8.9	119	38.7 ± 7.7	**<0.0001**
Appendicular lean mass (kg)	362	19.8 ± 4.8	124	17.2 ± 4.0	**<0.0001**
38% Tibia					
Ct. BMC (mg/mm)	326	314.7 ± 76.3	120	271.1 ± 80.3	**<0.0001**
Ct. Area (mm^2^)	327	261.8 ± 58.6	120	229.8 ± 60.7	**<0.0001**
CSA (mm^2^)	326	411.1 ± 72.3	120	389.8 ± 62.3	**0.005**
CSMI (mm^4^)	326	13063.7 ± 5241.0	120	10911.6 ± 4152.7	**0.0001**
SSI (mm^3^)	326	1686.8 ± 455.9	120	1489.4 ± 416.7	**<0.0001**
66% Tibia					
CSMA (mm^2^)	319	5368.8 ± 1131.3	118	4665.2 ± 1077.0	**<0.0001**
Muscle density (mg/cm^3^)	311	70.4 ± 2.8	117	68.5 ± 2.8	**<0.0001**
Grip strength (kg)	362	27.6 ± 9.3	126	20.1 ± 7.2	**<0.0001**
CRT					
Relative force (N/kg)	357	1.4 ± 0.3	126	1.3 ± 0.4	0.352
Relative power (W/kg)	357	8.0 ± 2.8	126	5.8 ± 1.7	**<0.0001**
Time per test (s)	357	4.7 ± 1.5	126	6.3 ± 1.9	**<0.0001**

All values are mean ± SD. Bold indicates *P* < 0.05.

BMC, bone mineral content; BMI, body mass index; BW, body weight; CRT, chair rise test; CSA, cross‐sectional area; CSMA, cross‐sectional muscle area; CSMI, cross‐sectional moment of inertia; Ct, cortical; m1LH, multiple one leg hop; SD, standard deviation; SSI, stress strain index; s2LJ, single two‐legged jump.

**Figure 1:**
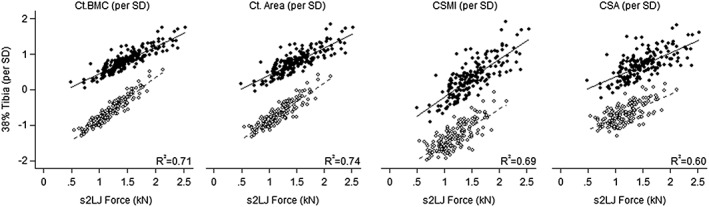
The relationship between s2LJ force with bone outcomes at the 38% tibia. Adjustments were made for weight and height. Black circles and solid line represent men; white circles and dashed line represent women. Ct, cortical; BMC, bone mineral content; CSA, cross‐sectional area; CSMI, cross‐sectional moment of inertia; s2LJ, single two‐legged jump.

The associations between muscle force and pQCT tibial bone measures are presented in *Table* 4. Women had greater positive associations between s2LJ/m1LH force and bone measures at the 38% tibia compared with men. In women, for every 1 kN increase in s2LJ force, there were positive differences in Ct.BMC by 26% (CI: 17.0, 35.6) and Ct.Area by 17% (CI: 9.2, 25.0) following adjustments (*Table* 4, *Figure* *1*). The main effect of s2LJ force was positively associated with CSMI, an estimate of bone bending strength (*P* < 0.0001) and CSA (*P* < 0.0001), with similar findings in m1LH force. The associations between muscle force and bone measures were markedly greater in women than in men (*Table* 4).

**Table 4:** Association between jump muscle force and bone outcomes at the 38% tibia in men and women

	Model 1			Model 2		
	Men	Women	*p*‐int	Men	Women	*p*‐int
**m1LH** (*n*=333)						
Ct. BMC (mg/mm)	16.0 (10.3, 21.6)	37.3 (29.4, 45.2)	**<0.0001**	7.4 (0.8, 14.1)	25.7 (16.4, 35.1)	**<0.0001**
Ct. Area (mm^2^)	16.0 (11.1, 20.8)	30.0 (23.1, 36.7)	**0.001**	6.6 (1.0, 12.2)	17.2 (9.3, 25.1)	**0.010**
CSMI (mm^4^)	30.6 (21.4, 40.0)	38.3 (25.3, 51.2)	0.347	9.1 (‐1.2, 19.5)	9.2 (‐5.4, 23.8)	0.998
CSA (mm^2^)	12.3 (7.6, 17.0)	10.4 (3.8, 17.0)	0.647	2.8 (‐2.5, 8.1)	‐2.3 (‐9.7, 5.2)	0.194
**s2LJ** (*n*=360)						
Ct. BMC (mg/mm)	16.1 (10.6, 21.6)	37.9 (30.4, 45.3)	**<0.0001**	7.8 (1.2, 14.4)	26.3 (17.0, 35.6)	**<0.0001**
Ct. Area (mm^2^)	15.5 (10.7, 20.3)	30.9 (24.4, 37.4)	**<0.0001**	5.7 (0.03, 11.4)	17.1 (9.2, 25.0)	**0.004**
CSMI (mm^4^)	28.4 (19.3, 37.4)	43.8 (31.6, 56.0)	**0.046**	8.1 (‐2.3, 18.5)	15.5 (0.9, 30.0)	0.317
CSA (mm^2^)	10.8 (6.2, 15.5)	13.3 (7.1, 19.5)	0.527	1.9 (‐3.4, 7.3)	1.1 (‐6.4, 8.6)	0.832

Data are expressed as percent increase in bone outcome per 1 kN increase in force. Values are beta‐coefficients with 95% confidence intervals. Bold indicates *P* < 0.05. Model 1: included a muscle force*sex interaction term, Model 2: <Model 1 plus weight and height adjustments. *p*‐int displays the *p*‐values from the sex*force interactions.

BMC, bone mineral content; CSA, cross‐sectional area; CSMI, cross‐sectional moment of inertia; Ct, cortical; m1LH, multiple one leg hopping; s2LJ, single two legged jump.

## Discussion

Our study showed that in ageing rural Gambians, the FNIH cut‐point for sarcopenia of appendicular lean mass of <19.75 kg in men and <15.02 kg in women was the most robust in identifying men and women with low functional ability in the upper and lower limb. In women, the FNIH aLM definition of sarcopenia was most discriminative, with good sensitivity and specificity, of low jump muscle power and force rather than low grip strength. Using the FNIH definition, 20% of men and 45% of women were classified as sarcopenic. Jump muscle force was positively associated with bone mineral content, cortical area, and bone cross‐sectional area and to a greater magnitude in women compared with men.

Although there have been various cut‐points developed for defining sarcopenia, there is no consensus as to which definition is most robust in identifying sarcopenic individuals from different ethnic groups. In our study, depending on which definition was used, the prevalence of sarcopenia differed quite significantly. However, the FNIH aLM cut‐point had the greatest specificity and sensitivity in detecting low functional ability in ageing Gambian men and women. Our finding is consistent with a study in Black women aged 45–84 years from the north‐west of South Africa.^31^ Also, the FNIH aLM definition identified a higher prevalence of sarcopenia in Gambian women (45%) when compared with the EWGSOP definition. In line with our findings, there was also a greater proportion of women classified as sarcopenic (39%) from the South African study according to the FNIH aLM definition,^31^ with a higher prevalence of obesity in the Black South African women compared with Gambian women.

Jumping mechanography has been shown to assess the effects of ageing with more sensitivity than traditional measures of functional ability.^25^, ^32^ In our study, jump muscle power and force best agreed with the sarcopenia definitions in identifying sarcopenic women and men with better sensitivity and specificity than grip strength. Studies have shown that older individuals who fall, have decreased muscle power in the lower limbs compared with their non‐faller counterparts.^11^, ^33^ These findings indicate that jumping mechanography may be a useful measure to incorporate into sarcopenia definitions than traditional tests. It should however be noted that there was moderate to good agreement between FNIH aLM and the functional measures. The significant odds ratios for FNIH and other sarcopenia definitions may also reflect the relationship between lean mass and function, rather than indicating the appropriateness of the measure of sarcopenia. Further work is needed to confirm our findings and to confirm the most appropriate cut‐offs.

Our findings show positive associations between jump force and BMC and size in older Gambian men and women; these were stronger in women than in men. In line with the theory that bone proportionally adapts to the loads from forces generated by muscle contractions,^14^ Gambian women may have enhanced sensitivity to applied force. The greater differences in muscle force in Gambian women may have had a greater effect on the amount of bone loss through endosteal resorption (indicated through cortical BMC and cortical area) and gained through periosteal apposition (greater bone CSA) compared with Gambian men. Women in this rural community remain active throughout ageing which may drive these adaptations in the bone.

There are potential limitations in this study. The observational design does not allow us to draw conclusions about causality or within individual change; however, this will be possible with the longitudinal data. The sex‐specific 20th percentile assumption for low muscle power and force was arbitrarily chosen because population norms for young adult Gambian men and women are not currently available; this may not apply the same way throughout due to generational lifestyle differences between older and younger Gambians. Jumping mechanography is a new approach to the measurement of functional capability and has been validated as a reproducible tool but not in ethnically diverse populations. The participants that were excluded in the ROC analyses because they were ‘unable’ to perform the s2LJ or m1LH were older, weighed less, and had a lower aLM than the participants who were ‘able’ to perform these tests. However, the significant correlation between CRT relative power and grip strength suggest that the FNIH definition will also hold true in the ‘unable’ group. One of the main strengths of this study is that it is the first and largest study in West Africa in which quantitative measurements of bone and muscle have been collected. Our suite of measurements are unique to sub‐Saharan Africa and allow detailed characterization of musculoskeletal health.

Our findings show that the FNIH aLM definition best identifies both sarcopenic Gambian men and women with low functional ability. As such, 20% of men and 45% of women aged ≥40 years were classified as sarcopenic, of which 81% of sarcopenic men and 68% of sarcopenic women were aged over 60 years. Muscle force was positively associated with tibial bone measures, which was far more pronounced in women than in men. In this population, determining methods to maintain muscle capability during ageing will be beneficial, as majority of sarcopenic Gambians were aged over 60 years. The different definitions of sarcopenia cannot be applied to all populations as there are significant ethnic differences in body composition effecting the components used in determining the prevalence of sarcopenia. Within 35 years, most of the older world population will be living in LMICs. Thus, it is imperative that the national health agendas of these countries prioritize and focus on sarcopenia and osteoporosis, to avoid the long‐term social, economic, and healthcare costs.

## Conflicts of interest

A.Z., L.M.J., A.P., and K.A.W. all declare no conflict of interest. P.R.E. reports grants and consulting fees from Amgen, Eli‐Lilly, and Gilead. C.C. reports consulting fees from Alliance for Better Bone Health, Amgen, Eli‐Lilly, GSK, Medtronic, Merck, Novartis, Pfizer, Roche, Servier, Takeda, and USB.

## Author Contribution

K.A.W., A.P., and L.M.J. designed and conducted the study; A.Z. and K.A.W. analysed the data; A.Z., K.A.W., A.P., C.C., and P.R.E. interpreted the data; A.Z., L.M.J., A.P., C.C., P.R.E., and K.A.W. prepared the manuscript and are responsible for the final content. All authors read and approved the final manuscript.
